# N^6^-Methyladenosine in Vascular Aging and Related Diseases: Clinical Perspectives

**DOI:** 10.14336/AD.2023.0924-1

**Published:** 2024-08-01

**Authors:** Chen Li, Le Liu, Shuang Li, You-Shuo Liu

**Affiliations:** ^1^Department of Geriatrics, The Second Xiangya Hospital, Central South University, Changsha, Hunan, China.; ^2^Institute of Aging and Age-related Disease Research, Central South University, Changsha, Hunan, China

**Keywords:** disease, vascular aging, N^6^-methyladenosine, RNA modification, senescence

## Abstract

Aging leads to progressive deterioration of the structure and function of arteries, which eventually contributes to the development of vascular aging-related diseases. N^6^-methyladenosine (m^6^A) is the most prevalent modification in eukaryotic RNAs. This reversible m^6^A RNA modification is dynamically regulated by writers, erasers, and readers, playing a critical role in various physiological and pathological conditions by affecting almost all stages of the RNA life cycle. Recent studies have highlighted the involvement of m^6^A in vascular aging and related diseases, shedding light on its potential clinical significance. In this paper, we comprehensively discuss the current understanding of m^6^A in vascular aging and its clinical implications. We discuss the molecular insights into m^6^A and its association with clinical realities, emphasizing its significance in unraveling the mechanisms underlying vascular aging. Furthermore, we explore the possibility of m^6^A and its regulators as clinical indicators for early diagnosis and prognosis prediction and investigate the therapeutic potential of m^6^A-associated anti-aging approaches. We also examine the challenges and future directions in this field and highlight the necessity of integrating m^6^A knowledge into patient-centered care. Finally, we emphasize the need for multidisciplinary collaboration to advance the field of m^6^A research and its clinical application.

## 1. Introduction

Aging leads to progressive deterioration of the structure and function of arteries. More specifically, aging induces intrinsic and extrinsic cellular changes that affect the phenotypes and behavior of building cells of the vascular wall, especially endothelial cells (ECs) and vascular smooth muscle cells (VSMCs). Macroscopically, aging vasculature presents dilated lumen, thickened wall, diffused stiffness, and impaired angiogenesis. These alterations seriously compromise proper tissue function and eventually contribute to the development of vascular diseases [1, 2]. Age-related macrovascular diseases, such as cardiovascular diseases (CVDs), are the leading cause of death and a major driver of disability in the elderly worldwide [3]. Aging-induced structural and functional changes in microvessels exert a pivotal role in the aging process of many organs and are one of the common denominators of various aging-related diseases, such as Alzheimer's disease (AD), vascular cognitive impairment, and kidney disease [4]. Understanding vascular aging and its molecular mechanisms is crucial for therapeutic approaches to age-related diseases.

**Figure 1.**
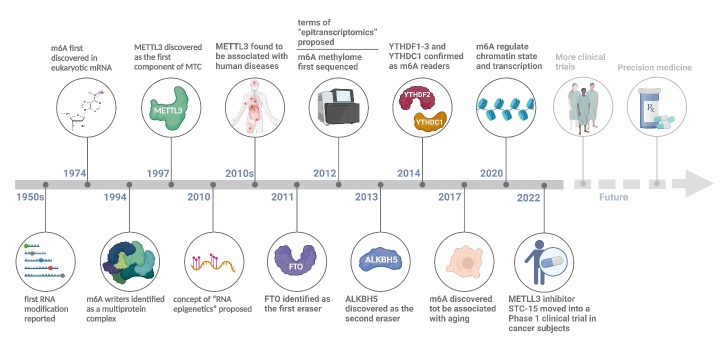
**Timeline of the discovery and research history of m^6^A modification**. Major findings in the m^6^A field are highlighted. This figure was created with the aid of Biorender (https://biorender.com/).

N^6^-methyladenosine (m^6^A) methylation, an emerging frontier in epigenetic research, involves the methylation at the N^6^ position of adenine in RNA. It has been found to play a significant role in vascular aging and related diseases [5-7]. To better learn about the discovery and research history of m^6^A in these diseases, we review the timeline of m^6^A (Fig. 1). RNA modifications have gained attention comparable to DNA and histone modifications in the field of epigenetics. Since the 1950s, over one hundred distinct RNA chemical modification types have been identified [8]. The discovery of m^6^A modification dates back to 1974 [9, 10]. m^6^A was identified as the most prevalent internal modification on messenger RNAs (mRNAs) in a diverse spectrum of eukaryotes (eg. yeast [11], plants [12], insects [13], mammals [9, 10, 14]), multiple viruses [15], and bacteria [16]. Nonetheless, due to the lack of powerful detective techniques to support in-depth studies on the distribution and function of m^6^A modification, limited progress was made during the following decades. In the 1990s, m^6^A methyltransferase was purified and characterized as a multicomponent protein complex along with the identification of methyltransferase-like 3 (METTL3) as one of its components [17, 18]. In 2011, fat mass and obesity-associated protein (FTO) was identified as the first m^6^A demethylase, revealing a reversible regulatory mechanism [19]. Hereafter, a series of other regulatory machineries of writers, erasers, and readers, have been identified [20]. The landscape of m^6^A modification at a transcriptome-wide level was first compiled by m^6^A-seq or methylated RNA immunoprecipitation sequencing (MeRIP-seq) in 2012 [21, 22]. m^6^A sites are non-randomly distributed in the transcriptome and are adjacent to consensus motif RRACH (R=A or G; H=A, C or U), enriched in the 3' untranslated region (3' UTR), near the stop codons, and within long internal exons [21, 22]. m^6^A is deposited in both coding and non-coding RNA (ncRNA) by m^6^A methyltransferases (writers), removed by demethylases (erasers), and recognized by binding proteins (readers) to affect almost all stages of RNA life cycle [20]. The robust technical advancements supporting extensive research on m^6^A and its function have catalyzed the emergence of a novel field of study known as “epitranscriptomics”. Thus far, an overwhelming number of reports have demonstrated the multifaceted effects of m^6^A on almost all major biological processes and human diseases (including vascular aging and related diseases) via expression regulation of various RNAs [23, 24]. Promisingly, recent studies start to focus on revealing novel therapies targeting m^6^A modification and their preclinical efficacy, heralding an era of translational research in this field [25].

In this paper, we will compile emerging evidence on the role of m^6^A methylation in vascular aging and highlight its potential clinical application in vascular aging-related diseases.

## 2. m^6^A Modification: Bridging Molecular Insights and Clinical Realities

### 2.1 Biological effects of m^6^A regulators

**Figure 2.**
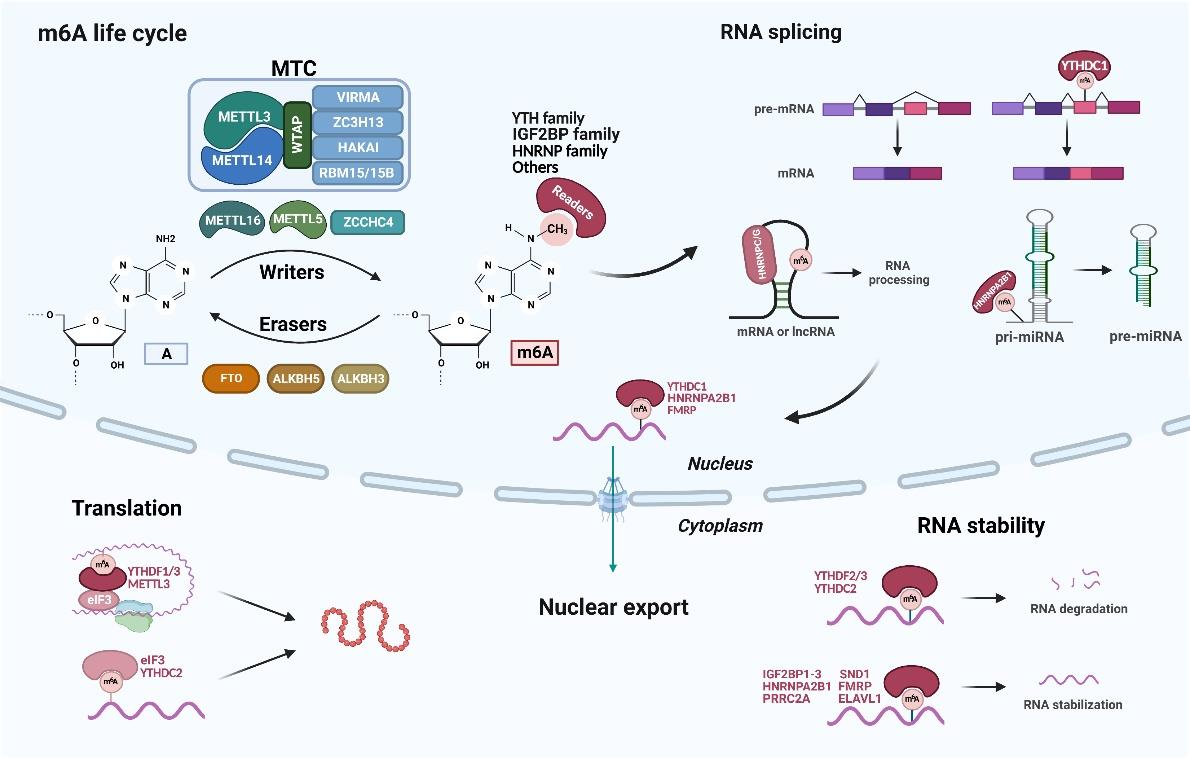
**The composition and function of m^6^A modification**. m^6^A methylation is a dynamic and reversible epigenetic modification that is installed by writers, removed by erasers, and recognized by readers. RNA m^6^A methylation can affect almost all stages of the RNA life cycle, including RNA splicing, maturation, nuclear export, degradation, stabilization, and translation. This figure was created with the aid of Biorender (https://biorender.com/).

As mentioned above, m^6^A modification is a reversible process where m^6^A is installed by writers, removed by erasers, and recognized by readers (Fig. 2). Moreover, m^6^A can regulate RNA fate and metabolism (e.g., splicing, maturation, nuclear export, degradation, stabilization, and translation), which in turn, underlies their critical role in a myriad of physiological and pathological processes [26-28].

#### 2.1.1 Writers

m^6^A methylation is catalyzed by m^6^A methyltransferase complex (MTC) [17] that consists of a key component, METTL3/METTL14 dimer, and cofactors such as Wilms tumor 1 associated protein (WTAP), vir-like m^6^A methyltransferase-associated protein (VIRMA, also called KIAA29), RNA-binding motif protein 15/15B (RBM15/15B), zinc finger CCCH-type containing 13 (ZC3H13), and HAKAI (also called CBLL1) [29-34]. METTL3, the first-identified MTC component, is the only subunit with catalytic activity in MTC that catalyzes the transfer of methyl groups from S-adenosylmethionine (SAM) to adenine in RNA [18, 35-37]. METTL14 is the other core part in MTC that unites with METTL3 forming a stable heterodimer [29]. Unlike METTL3, METTL14 contains a degenerate active site and presents non-catalytic effects on structurally supporting the catalytic function of METTL3 as well as substrate recognition [35-37]. WTAP serves as a regulatory subunit that interacts with METTL3 and METTL14 to recruit them into nuclear speckles, enhancing the catalytic activity of m^6^A methyltransferase. In addition, WTAP and METTL3 regulate genes associated with transcription and RNA processing [30]. VIRMA directs METTL3/METTL14/ WTAP to mediate region-selective mRNA methylation in 3'UTR and near stop codon [38]. RBM15/RBM15B recruits MTC to specific RNA sites, which leads to the methylation of adenosine in neighboring consensus motifs [32]. ZC3H13 can bridge RBM15 to WTAP [34] and is necessary for the nuclear localization of MTC [33]. HAKAI, a recently identified MTC component, is needed for stabilizing other constituents of MTC through its ubiquitination domain [39].

Despite large research efforts on the role of MTC in mRNA m^6^A modification, there is evidence revealing other m^6^A writers (METTL16, METTL5, and ZCCHC4) to catalyze m^6^A methylation of a subset of RNAs [40-42]. METTL16 is reported to mediate m^6^A modification in U6 small nuclear RNA (snRNA) and *MAT2A* mRNA [42, 43]. ZCCHC4 is the enzyme responsible for m^6^A modification of 28S ribosomal RNA (rRNA) and some mRNAs [40, 41]. METTL5 exerts m^6^A methyltransferase function on 18S rRNA by forming a heterodimer with methyltransferase activator TRMT112 [41].

#### 2.1.2 Erasers

The demethylation of m^6^A modification in RNA is mediated by erasers including FTO, AlkB homolog 5 (ALKBH5), and ALKBH3. FTO is the first identified m^6^A demethylase [19]. In addition to m^6^A demethylation, FTO has been proven to catalyze other patterns of RNA modifications, such as N^6^,2'-O-dimethyladenosine (m^6^A_m_) and N^1^-methyladenosine (m^1^A) [44-46]. ALKBH5 is another established m^6^A eraser. Unlike FTO, ALKBH5 appears a specific role in m^6^A [47]. Moreover, recent studies have discovered a new m^6^A demethylase, ALKBH3, which participates in the demethylation of tRNA [48]. The enzymatic activity of these erasers relies on their oxidative function in a Fe(II)/2-oxoglutarate (2-OG)-dependent manner [49].

#### 2.1.3 Readers

Writers and erasers mediate the deposition and removal of m^6^A, respectively. However, it is readers that recognize m^6^A and determine different fates of RNA after modification, including but not limited to YT521-B homology (YTH) domain family, splicing factor heterogeneous nuclear ribonucleoproteins (HNRNP) family, insulin-like growth factor 2 mRNA binding protein (IGF2BP) family.

Different readers present different biological roles [50]. The YTH domain family contains YTH domain family protein 1-3 (YTHDF1-3) and YTH domain containing protein 1-2 (YTHDC1-2). The canonical model considers that YTHDF1 enhances mRNA translation [51]; YTHDF2 accelerates mRNA decay [52]. YTHDF3 exerts dual functions to promote translation and strengthen degradation through cooperation with YTHDF1 and YTHDF2, respectively [53, 54]. However, contradictory results have been observed that the functions of YTHDFs are redundant [55]. YTHDC1 regulates mRNA splicing [56] and nuclear export [57]. YTHDC2 has been revealed to elevate translation efficiency and reduce abundance of target mRNA [58]. HNRNP family includes HNRNPA2B1, HNRNPC, HNRNPG. HNRNPA2B1 is an m^6^A reader regulating multiple processes of RNA metabolism such as primary microRNA (pri-miRNA) processing, alternative splicing [59], nucleocytoplasmic trafficking [60], and stabilization [61]. m^6^A leads to RNA allostery to increase the accessibility of HNRNPC and HNRNPG, a phenomenon termed the "m^6^A switch", which can affect mRNA expression and alternative splicing [62, 63]. IGF2BP family members, IGF2BP1-3, have been reported to enhance the stability of target mRNA through recognition of m^6^A [64].

**Figure 3.**
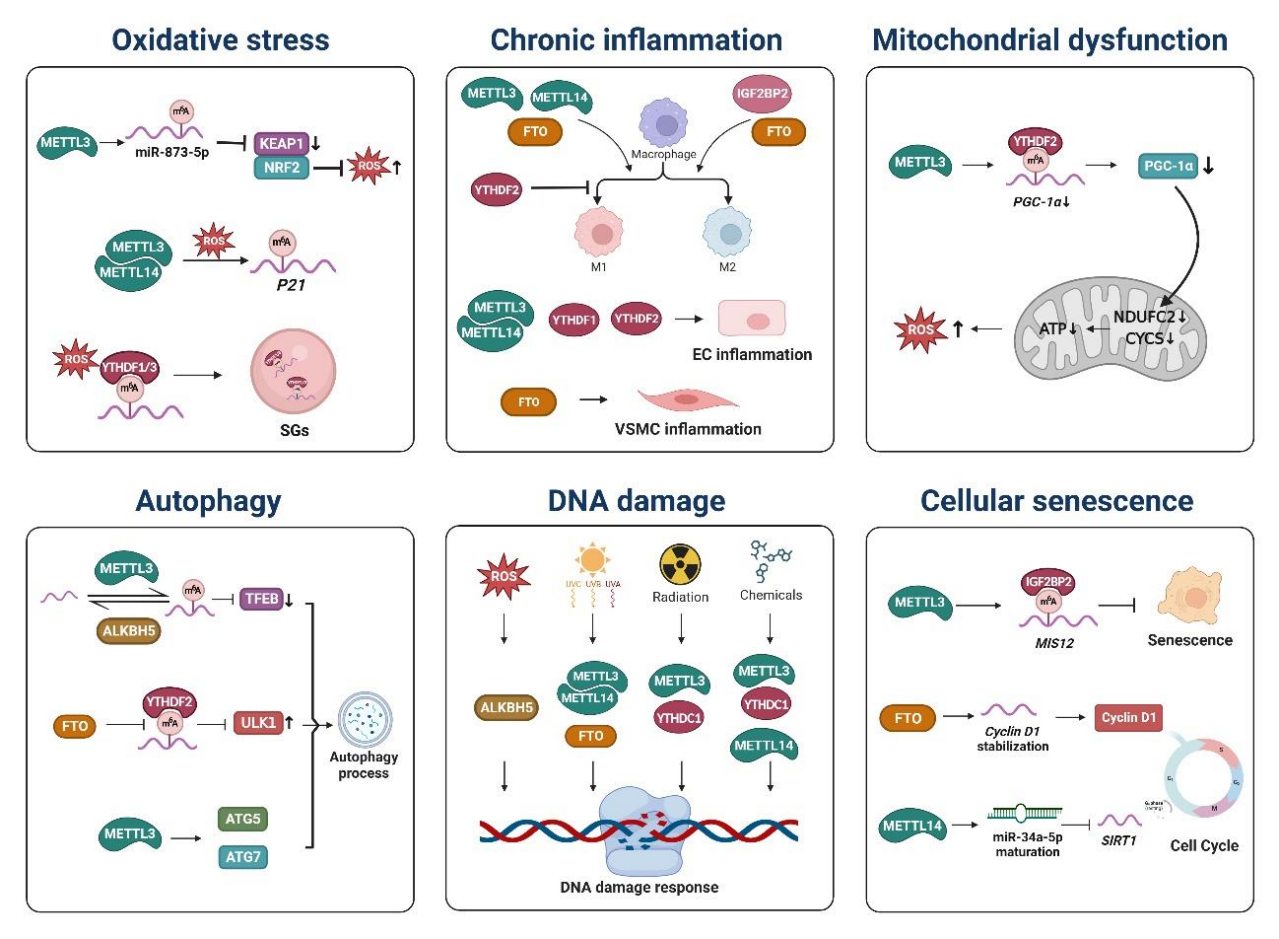
**Potential regulatory mechanisms of m^6^A modification in vascular aging**. The aberrant m^6^A modification participates in various molecular and cellular mechanisms that lead to vascular aging, including oxidative stress, chronic inflammation, mitochondrial dysfunction, autophagy, DNA damage, and cellular senescence. This figure was created with the aid of Biorender (https://biorender.com/). ROS: reactive oxygen species; SG: stress granule; EC: endothelial cell; VSMC: vascular smooth muscle cell.

In addition to the three aforementioned well-studied protein families, several other readers have been reported. Fragile X mental-retardation protein (FMRP) has been demonstrated to modulate m^6^A-dependent mRNA nuclear export [65] and stability [66]. Recent studies have identified other readers that stabilize mRNA through m^6^A modification, including proline-rich coiled-coil 2A (PRRC2A) [67], ELAV-like RNA binding protein 1 (ELAVL1) [68, 69], and staphylococcal nuclease and tudor domain containing 1 (SND1) [70]. Another m^6^A reader, eukaryotic translation initiation factor 3 (eIF3), can promote translation by binding 5’-UTR m^6^A [71]. METTL3 located in nuclear speckles serves as methyltransferase as mentioned before. Interestingly, in contrast to its nuclear existence as an m^6^A writer, cytoplasmic METTL3 acts as a potential m^6^A reader instead. Mechanistically, METTL3 promotes mRNA translation through interaction with eIF3h to form RNA looping [72].

### 2.2 Potential regulatory mechanisms of m^6^A modification in vascular aging

Vascular aging is a complicated biological process driven by intertwined cellular and molecular mechanisms (such as oxidative stress, chronic inflammation, mitochondrial dysfunction, autophagy, DNA damage, and cellular senescence) contributing to various aging-related diseases in major organs [4]. m^6^A methylation is well-documented to modulate the expression of target genes involved in these mechanisms and therefore may apply pleiotropic activities on vascular aging [73-77]. The interplay between m^6^A modification and key pathways implicated in vascular aging bridges molecular insights with clinical realities, highlighting its significance in understanding disease pathogenesis (Fig. 3).

#### 2.2.1 Oxidative stress

Oxidative stress is an imbalance between pro-oxidation and anti-oxidation systems, resulting in the overproduction of reactive oxygen species (ROS), which can contribute to various diseases by damaging cells and tissues [78]. Oxidative stress is considered one of the main determinants in vascular aging and related diseases [79, 80]. Aging vasculature produces excessive ROS, primarily generated by reduced nicotinamide adenine dinucleotide phosphate (NADPH) oxidase, mitochondrial respiratory chain, xanthine oxidase, and uncoupled endothelial nitric oxide (NO) synthase [81]. The overproduction of ROS leads to endothelial dysfunction and large artery stenosis [82-84]. Adequate production of NO by the vascular endothelium exerts pivotal roles in regulating vasodilation and tissue perfusion [85], while compromised bioavailability of endothelium-derived NO is critical for mediating the effects of oxidative stress in blood vessel function with aging [82, 86]. Additionally, the weakening antioxidant response mediated by nuclear factor erythrocyte 2-related factor 2 (NRF2) and the inactivation of mitochondrial manganese superoxide dismutase (SOD2) also result in chronic oxidative stress in the aging vasculature [87, 88]. It has demonstrated that through regulating Kelch-like ECH-associated protein 1 (KEAP1)/NRF2 signaling, METTL3/m^6^A/miR-873-5p axis mitigates oxidative stress and cell apoptosis [89].

ROS induces cell senescence along with altered m^6^A level and expression profiles of m^6^A modification regulators and their target genes [90]. Further, METTL3/METTL14-catalyzed m^6^A has been revealed to promote *P21* expression, thus aggravating oxidative stress-induced cellular senescence [91]. These studies indicate the potential role of m^6^A in age-related pathophysiological changes induced by oxidative stress.

Under oxidative stress, mRNAs are additionally m^6^A-modified in the 5' UTR, facilitating mRNA triaging to stress granules (SGs), which are membraneless granules mainly containing untranslated mRNAs and RNA-binding proteins in cells in response to stress conditions [92]. YTHDF proteins, especially YTHDF1/3 are crucial in regulating SG formation [92, 93]. These results suggest a novel function of m^6^A modification in regulating the oxidative stress response.

#### 2.2.2 Chronic inflammation

Inflammation is a protective response to various internal and external stimuli such as injury, infection, and tissue stress that may challenge homeostasis; however, persistent, or excessive inflammation can cause tissue injury and diseases [94]. “Inflamm-aging” is a result of an imbalance between inflammatory and anti-inflammatory networks, and represents the chronic, low-grade, non-infective inflammation that develops with age [95]. Importantly, this sterile inflammation has been regarded as a dominator of vascular aging and a central player in most vascular aging-related diseases [96-98]. Several anti-inflammatory interventions such as canakinumab and colchicine have been proven effective in the context of some vascular aging-related afflictions [99]. Emerging evidence has unraveled the role of m^6^A modification in the modulation of inflammation [100].

Increased abundance and changed polarization of macrophages are important hallmarks of aging [101]. Recent studies have established that m^6^A modification alters when stimulated by oxidized low-density lipoprotein (oxLDL) [102, 103], lipopolysaccharide (LPS) [104], or interferon-γ (IFN-γ) [105], or IL-4 [106] in macrophages, and therefore dynamically regulating macrophage inflammation and polarization. Literature has described that m^6^A participates in the modulation of macrophage polarization through targeting signal transducer and activator of transcription 1 (*STAT1*) [105, 107], and myeloid differentiation primary response 88 (*MYD88*) [104]. Emerging evidence has established that m^6^A regulators, METTL3 [107], METTL14 [104], FTO [108], IGF2BP2 [106], and YTHDF2 [105] are involved in the modulation of macrophage polarization. Specifically, METTL3 [107] and METTL14 [104] promote macrophage M1 polarization, while IGF2BP2 is a positive regulator of M2 activation [106]. Downregulation of FTO suppresses both M1 and M2 macrophage polarization [108].

In addition to the regulation of macrophage inflammation, m^6^A also plays an important role in EC and VSMC inflammation. For example, METTL3- and METTL14-mediated m^6^A modification promotes EC inflammation through regulating NOD-like receptor protein 1 (*NLRP1*), Krüppel-like factor 4 (*KLF4*), or forkhead box O1 (*FOXO1*) mRNA [109, 110]. Moreover, downregulation of FTO expression suppresses VSMC inflammation by targeting nuclear receptor subfamily 4, group A, member3 (*NR4A3*) [111].

An in-depth exploration of the interplay between m^6^A and inflammation is of great importance to identify more pathogenic pathways and develop promising therapeutic approaches to inflammation-associated diseases.

#### 2.2.3 Mitochondrial dysfunction

Dysfunctional mitochondria underly the pathological mechanism of vascular aging [83]. Structural and functional alterations in mitochondria potentially result in EC and VSMC senescence, including dysfunctional mitochondrial dynamics, mitochondrial energy metabolism, mitophagy, and consequent mitochondrial DNA mutations [112-114]. Particularly, excessive ROS produced by dysfunctional mitochondria exert a pivotal role in vascular aging as described above. Additionally, numerous interplays exist between mitochondrial dysfunction and other age-related molecular and cellular mechanisms, such as low-grade inflammation and telomere attrition, which further exacerbate vascular aging [114].

Emerging evidence suggests the regulatory function of m^6^A modification in mitochondrial homeostasis. Specifically, METTL3 coordinates with YTHDF2 to enhance oxLDL-induced mitochondrial dysfunction and inflammation in monocytes by promoting m^6^A-mediated mRNA degradation of peroxisome proliferator-activated receptor gamma coactivator 1-alpha (*PGC-1α*), a key regulator of mitochondrial biogenesis [115]. METTL3 and YTHDF2 synergistically downregulate electron transport chain proteins cytochrome c (CYCS) and NADH: ubiquinone oxidoreductase subunit C2 (NDUFC2), and diminish ATP generation and oxygen consumption, and thus elevate ROS production [115]. These results provide a potential mechanism for vascular aging-related diseases.

#### 2.2.4 Autophagy

Autophagy serves as a highly conserved intracellular degradation and recycling mechanism of senescent or malfunctioning organelles to maintain cellular renovation and homeostasis. Notably, it is believed that autophagy is a fundamental mechanism for the maintenance of cardiovascular homeostasis during aging [116]. Experiments demonstrated that autophagy-deficient EC appears increased oxidative stress, impaired NO bioavailability, and increased inflammatory mediators. Autophagic activity is decreased in vascular tissues of aged humans and mice [117]. Dysregulation of autophagic processes is associated with vascular aging and related diseases [118]. The aspects of arterial aging can be reversed with the administration of autophagy enhancers such as spermidine [119] and nicotinamide mononucleotide [120].

Recent studies have shown that m^6^A modification plays important roles in autophagy regulatory networks by regulating autophagy-related genes, such as transcription factor EB (*TFEB*) [121], unc-51-like kinase 1 (*ULK1*) [122], and autophagy-related (*ATG*) genes [123, 124], which serves as a potential mechanism in vascular aging. For example, METTL3 and ALKBH5 reversely regulate m^6^A modification of *TFEB* to affect autophagic flux [121]. FTO potentiates the initiation of autophagy through increasing *ULK1* mRNA stability by demethylation [122]. Notably, the m^6^A writer METTL3 has been identified as a regulator of autophagy to affect VSMC behavior, serving as a potential mechanism of vascular aging. Specifically, METTL3 promotes autophagosome formation by upregulating the expression of *ATG5* and *ATG7*, and thus inhibits VSMC proliferation and prevents VSMCs from switching to synthetic phenotype [124]. Further studies are needed to fully elucidate the mechanisms underlying the role of m^6^A modification in autophagy and its potential implications for vascular aging-related diseases.

#### 2.2.5 DNA damage

DNA damage refers to the structural alteration of DNA caused by various endogenous and exogenous factors such as oxidation, replication errors, radiation, and chemical compounds, which can affect its normal function [125]. DNA damage has been implicated in the pathogenesis of vascular aging. It is reported that mice with genomic instability present increased vascular stiffness, vasodilator dysfunction, and blood pressure, suggesting the association between variations in human DNA repair genes and vascular aging [126]. Moreover, accumulating evidence indicates that DNA damage gives rise to endothelial dysfunction, inflammation, and cellular senescence, which all are hallmarks of vascular aging [4, 127].

Recent studies clarified the correlation between m^6^A and DNA damage and repair [128, 129]. Dysregulation of m^6^A modification has been implicated in the development of genomic instability, which can contribute to the onset and progression of various diseases, including aging-related disorders [76]. Recent studies have shown that m^6^A modification and its regulators play a critical role in the DNA damage response (DDR) pathway. Specifically, m^6^A regulators, such as METTL3 [130-132], METTL14 [132, 133], FTO [132], ALKBH5 [134], YTHDC1 [131], regulate DDR pathway through m^6^A in response to different stimuli such as ROS [134], ultraviolet [132], radiation [131], and various chemicals [130, 131, 133]. For example, METTL3-catalyzed m^6^A modification promotes DDR by stabilizing mRNAs of DNA damage repair factors, RAD51 recombinase (*RAD51*) and X-ray repair cross-complementing 5 (*XRCC5*), thereby maintaining cell survival [130]. Moreover, DNA damage repair has recently been verified to be closely related to m^6^A-modified retrotransposable element (RTE) RNAs, especially intronic long interspersed element-1 (LINE-1), which often inhibit hosting gene transcription and preferentially reside in host genes with vital functions in DNA damage repair [135]. METTL3-m^6^A-YTHDC1 axis facilitates homologous recombination-mediated repair by promoting the accumulation of DNA-RNA hybrids at double-strand break sites [131].

These findings suggest that m^6^A modification plays a crucial role in regulating DNA damage and repair processes, contributing to the development of aging and age-related diseases. However, further investigation is needed to explore the role and mechanisms of m^6^A modification in vascular aging through DNA damage.

**Table 1:** The role of m^6^A in vascular aging-related diseases.

Disease	m^6^A regulator	Target genes	Function	Ref
***Vascular diseases***				
**AS**	METTL3	*NLRP1*↑, *KLF4*↓	Mediates proatherogenic inflammatory responses in ECs induced by disturbed blood flow	[109]
	METTL3	*NPC1L1*↑	Promotes EC dysfunction and AS development	[152]
	METTL3	*JAK2*↑	Promotes oxLDL-stimulated EC dysfunction and AS progression	[153]
	METTL3	*STAT1*↑	Enhances oxLDL-induced inflammation in macrophages	[102]
	METTL3	*BRAF*↑	Facilitates macrophage inflammatory response and AS	[158]
	METTL3	pri-miR-375-3p↓	Promotes AS progression and destabilizes AS plaques by facilitating oxLDL-induced phenotypic transformation of VSMCs	[160]
	METTL3	*EGFR*↓	Attenuates endothelial atherogenic progression	[156]
	METTL14	*FOXO1*↑	Induces endothelial inflammation and contributes to AS progression	[110]
	METTL14	pri-miR-19a↓	Promotes the proliferation and invasion of atherosclerotic vascular ECs	[154]
	METTL14	*P65*↑	Decreases EC viability and enhances EC apoptosis stimulated by oxLDL	[155]
	METTL14	*MYD88*↑	Mediates macrophage inflammation and development of AS plaques	[104]
	METTL14	*UCHL5*↑	Exacerbates AS and VSMC phenotypic switching	[161]
**Hypertension**	FTO	*NR4A3*↑	DHA attenuates AngII-induced VSMC proliferation and inflammation by downregulating FTO expression	[111]
**AAD**	METTL3	pri-miR-34a↓	Promotes the formation of aortic aneurysm in mice	[175]
	METTL3	*SLC7A11*↓, *FSP1*↓	Promotes ferroptosis of VSMCs	[177]
	METTL3-METTL14 complex	*RIP3*↑	Promotes necroptosis and inflammation of VSMCs and aortic aneurysms progression	[179]
	KIAA1429, ALKBH5	pri-miR-143-3p↓	Oppositely affect aortic dissection progression through modulating VSMC proliferation	[180]
	FTO	*KLF5*↑	Mediates AngII-induced VSMC proliferation and migration	[176]
***Heart diseases***				
**MI**	METTL3	circ_0029589↓	Mediates IRF-1-induced macrophage pyroptosis and inflammation	[187]
	METTL3	pri-miR-503↓	Evokes miR-503 biogenesis in ECs; exosomal miR-503 triggers mitochondrial dysfunction and cardiomyocyte death	[188]
	METTL3	pri-let-7e↓, pri-miR-17-92↓	Improves post-ischemic neovascularization in MI mice	[191]
	METTL3	*SMAD2/3*↑	Aggravates MI-induced cardiac fibrosis through the activation of cardiac fibroblasts	[193]
	ALKBH5	*YTHDF1*↑	Reduces infarct size, improves cardiac function, and enhances cardiomyocyte proliferation	[189]
	ALKBH5	*SPHK1*↑	Maintains EC angiogenesis during acute ischemic stress	[190]
**HF**	METTL3	*MAP3K6*↑, *MAP4K5*↑, *MAPK14*↑	Controls cardiac homeostasis and hypertrophy	[183]
	FTO	*SERCA2a*↑	Alleviates ischemia-induced decrease in cardiac function	[182]
	FTO	-	Mice with cardiomyocyte-specific knockout of FTO presents worsened cardiac function	[197]
	FTO	*PGAM2*↑	Mitigates cardiac dysfunction in HF mice through modulating glycolysis	[199]
	FTO	-	FTO overexpression counteracts exercise benefits in HFpEF mice by inducing myocyte apoptosis, myocardial fibrosis, and myocyte hypertrophy	[201]
***Encephalopathy***				
**Stroke**	METTL3	pri-miR-335↓	Induces stress granule formation and attenuates the apoptosis of injury neuronal cells	[209]
	FTO	-	Minimizes poststroke brain damage and neurobehavioral deficits	[211]
	FTO	*PLPP3*↑	Mediates circSCMH1-promoted vascular repair after stroke	[212]
	YTHDC1	*PTEN*↓	Attenuates post-ischemic brain injury	[210]
	YTHDF1	*P65*↑	miR-421-3p presents anti-inflammatory effects in cerebral IRI by targeting YTHDF1	[213]
**AD**	METTL3	*Cyclin D2*↓	METTL3 knockout in the hippocampus leads to memory deficits, synaptic loss, and neuronal death	[220]
	METTL3	*STUB1*↑	Facilitates autophagic clearance of p-Tau in AD cell model	[221]
	METTL3	*ARC*↑	Rescues Aβ-stimulated decrease in ARC expression	[223]
	METTL3	*DNMT3A*↑	METTL3 ablation in monocyte-derived macrophages attenuates AD pathology	[224]
	FTO	-	Conditional knockout of FTO in the neurons decreases cognitive deficits in AD mice	[227]
	HNRNPA2B1	-	Serves as a connector between oTau and m^6^A-modified RNAs, subsequently regulating stress response and mediating the development of tauopathy	[228]
	IGF2BP2	-	IGF2BP2-related gene modules are significantly enriched in AD-associated biological processes	[229]
***Kidney diseases***				
**CKD**	METTL3	pri-miR-21↓	Promotes renal fibrosis by regulating inflammation	[235]
	METTL3	lncRNA MALAT1↑	Promotes TGF-β1-induced renal fibrosis	[236]
	METTL14	*Klotho*↓	Motivates VSMC osteogenic conversion stimulated by indoxyl sulfate	[242]
	WTAP	*NLRP3*↑	Induces cell pyroptosis and inflammation in DN models	[238]
	FTO	lncRNA GAS5↓	Promotes renal epithelial-mesenchymal transition and inflammation response	[237]
	FTO	*SOCS1*↑	Overexpression of FTO attenuates inflammation response and kidney injury of DN	[239]

The upward arrow represents “upregulated”; the downward arrow represents “downregulated”.

#### 2.2.6 Cellular senescence

Cellular senescence is a state of irreversible growth arrest that occurs after a finite number of divisions or in response to diverse stimuli, where cells present distinctive phenotypic switching [136]. Senescent cells accumulate in tissues during aging and contribute to the development of age-related diseases, including vascular aging. Evidence suggests that chronic senolytic treatment targeting senescent cells can attenuate aspects of vascular aging [137]. Senescent cells secrete senescence-associated secretory phenotype (SASP), including pro-inflammatory cytokines, chemokines, growth factors, and proteases. These SASP components drive autocrine and paracrine signaling, impairing vascular function, and promoting the development of vascular aging-related diseases such as atherosclerosis (AS) [76, 138].

Several studies have reported that alterations in m^6^A levels or in the expression of m^6^A regulators are associated with cellular senescence [91, 139, 140]. Emerging evidence further demonstrated that m^6^A regulators play a key role in cell senescence [77]. For example, METTL3/14 levels gradually decrease in cells experiencing replicative senescence, while the overexpression of METTL14 mitigates both replicative and premature senescence [140]. Similarly, METTL3 overexpression reverses the premature senescence of human mesenchymal stem cells (MSC) through m^6^A-mediated *MIS12* mRNA stabilization in cooperation with IGF2BP2 [141]. However, in another study, METTL3 was found to promote autophagy-regulated senescence in fibroblast-like synoviocytes [123]. Notably, researchers unraveled that METTL14 and FTO affect cell cycle to regulate senescence through m^6^A methylation. METTL14 regulates m^6^A-mediated maturation of miR-34a-5p, which promotes cell cycle arrest and senescence by targeting Sirtuin-1 (*SIRT1*) [142]. Another study revealed that FTO depletion increases m^6^A-mediated degradation of *Cyclin D1* mRNA, contributing to impaired cell cycle progression [143].

Recently, FTO and YTHDF2 have been found to be related to EC senescence, suggesting a potential mechanism of vascular aging. Li et al. reported the role of FTO in promoting EC senescence [144]. The results suggest that targeting m^6^A regulators or their target genes may represent a promising strategy for preventing or treating cellular senescence and its associated pathologies. Further studies are needed to fully elucidate the mechanisms underlying the role of m^6^A modification in cellular senescence and its potential implications for therapeutic interventions in vascular aging.

## 3. Unraveling Vascular Aging: Clinical Relevance

**Figure 4.**
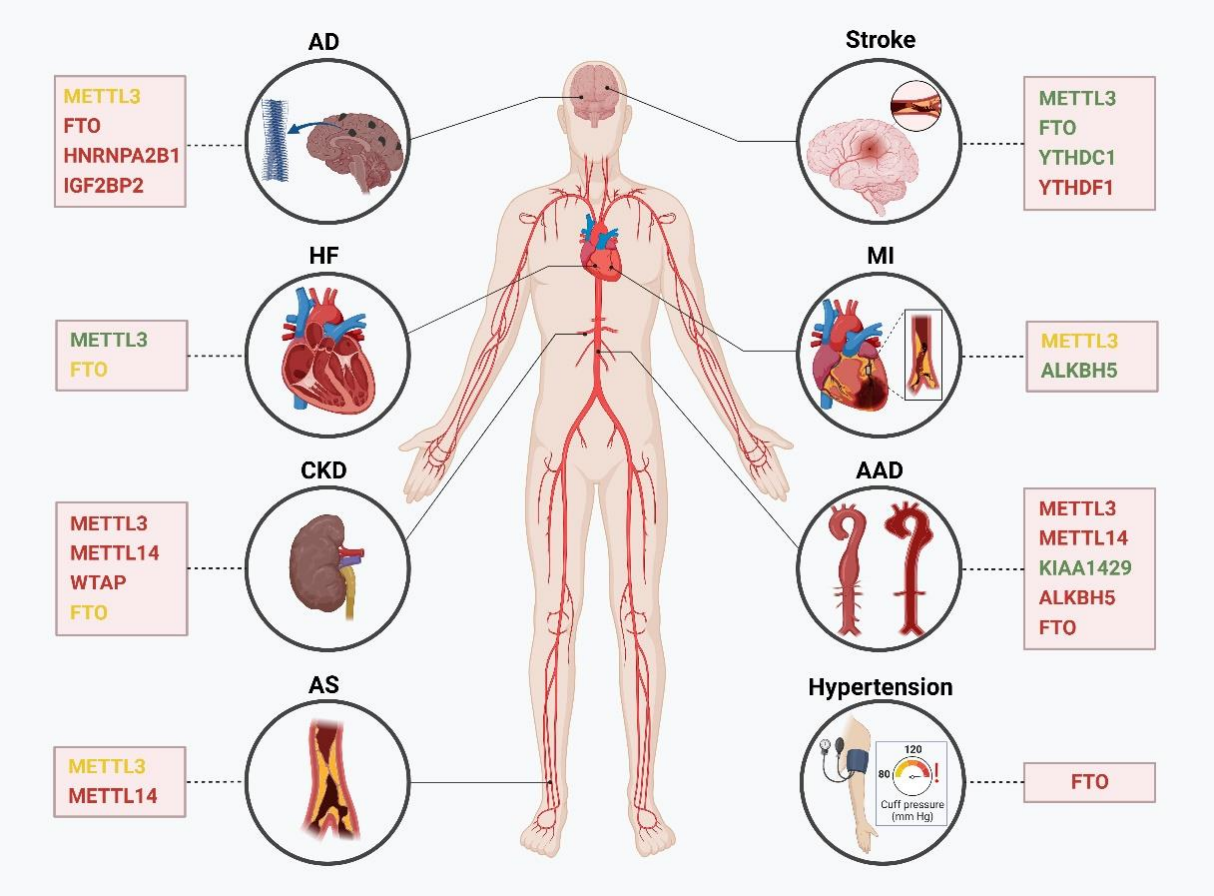
**Dysregulation of m^6^A modifiers in vascular aging-related diseases**. Red-colored modifiers indicate a pathogenic role, green-colored modifiers indicate a protective role, while yellow-colored ones have controversial roles reported, in the specific disease type. This figure was created with the aid of Biorender (https://biorender.com/). AD: Alzheimer's disease; HF: heart failure; MI: myocardial infarction; CKD: chronic kidney disease; AAD: aortic aneurysm/dissection; AS: atherosclerosis.

Vascular aging significantly impacts human health, leading to an increased risk of diseases. Identifying clinical manifestations associated with vascular aging allows for early detection and intervention. Accumulating evidence suggests that m^6^A presents pleiotropic effects on these diseases through gene expression regulation (Table 1). In this section, the role of m^6^A modification in different vascular aging-related diseases is explored, including CVDs, encephalopathy, and chronic kidney disease (CKD) (Fig. 4).

### 3.1 The roles of m^6^A in aging-related vascular diseases

Aging is the major risk factor for vascular diseases [145]. Marked aging changes in the vasculature make arteries more susceptible to vascular diseases. Recent research highlights the importance of m^6^A modification in modulating gene expression and maintaining vascular health during aging, while the dysregulation of m^6^A methylation may contribute to vascular dysfunction and age-related vascular diseases, such as AS, hypertension, and aortic aneurysm/dissection (AAD).

#### 3.1.1 m^6^A in AS

AS, a primary cause of CVDs, is an inflammatory disease that occurs in the large arteries [146]. Atherosclerotic plaques are characterized by accumulated and transformed lipids, macrophages, VSMCs, and necrotic cell debris in the subendothelial space just underneath the EC layer in the artery wall [101]. Vascular aging promotes the occurrence and development of AS, which in turn accelerates the process of vascular aging. Interestingly, altered levels of m^6^A and its regulators are identified in AS tissues, which indicates the potential interplay between m^6^A and AS [147, 148]. Notably, accumulating data have revealed the regulatory roles of m^6^A modification in the initiation and progression of AS through regulating EC dysfunction and inflammation, macrophage inflammation, and VSMC phenotypic transformation [149, 150].

EC dysfunction and inflammation are key pathological features in AS [151]. It is reported that METTL3-catalyzed m^6^A modification mediates proatherogenic inflammatory responses in ECs induced by disturbed blood flow through upregulating *NLRP1* and downregulating *KLF4* expression [109]. Further, METTL3-dependent m^6^A methylation of Niemann-Pick C1-Like 1 (*NPC1L1*) promotes EC dysfunction and AS development, possibly through regulating the mitogen-activated protein kinase (MAPK) pathway [152]. On the contrary, silencing METTL3 attenuates oxLDL-stimulated EC dysfunction and impedes AS progression *in vivo* by repressing the JAK2/STAT3 pathway via m^6^A/IGF2BP1-dependent regulatory mechanisms [153]. METTL14-mediated m^6^A modification also regulates the pathogenesis of AS by targeting ECs. Specifically, METTL14 facilitates the translation of *FOXO1* mRNA through YTHDF1 recognition in an m^6^A-dependent manner, which elevates adhesion molecule expression, induces endothelial inflammation, and ultimately contributes to the progression of AS [110]. Besides, METTL14 promotes the proliferation and invasion of atherosclerotic vascular ECs, probably through m^6^A-mediated pri-miR-19a processing [154]. METTL14 decreases EC viability and enhances EC apoptosis stimulated by oxLDL through m^6^A modification of *P65*; while knockdown of METTL14 can suppress AS progression *in vivo* [155]. On the other hand, m^6^A can also exert protective effects on AS. Mechanistically, METTL3 attenuates endothelial atherogenic progression through m^6^A-dependent mRNA decay of epidermal growth factor receptor (*EGFR*), a molecule related to EC dysfunction [156].

In addition to ECs, macrophages are pivotal players in the vascular inflammatory process of AS [157]. METTL3 and METTL14-mediated m6A modification affects AS process by regulating macrophage inflammation. Specifically, METTL3 facilitates the m^6^A modification of *STAT1* and v-Raf murine sarcoma viral oncogene homolog B (*BRAF*) to enhance oxLDL-induced inflammation in macrophages, thus promoting AS progression [102, 158]. METTL14 mediates macrophage inflammation and development of AS plaques via m^6^A-modified mRNA stabilization of *MYD88* that regulates the nuclear factor kappa B (NF-κB)/IL-6 signaling [104]. The RNA binding protein Matrin-3 (MATR3) inhibits oxLDL-induced macrophage inflammation and attenuates AS development by promoting the formation of METTL3-METTL14 complex and m^6^A-mediated mRNA decay of *MAPK* [103].

It is well-documented that the phenotypic transformation of VSMCs to proliferative synthetic cells contributes to AS development [159]. Recent evidence has revealed the role of m^6^A in this process. Specifically, silencing METTL3 attenuates AS progression and stabilizes AS plaques by mitigating oxLDL-induced phenotypic transformation of VSMCs partly through m^6^A-modified pri-miR-375 processing [160]. Moreover, METTL14 recruits YTHDF1 to enhance m^6^A and expression level of Ubiquitin C-terminal hydrolase L5 (*UCHL5*), which exacerbates AS and VSMC phenotypic switching by stabilizing the NLRP3 inflammasome [161].

#### 3.1.2 m^6^A in hypertension

Vascular aging is a crucial player in the pathogenesis of hypertension. Hypertension has become a crucial risk factor for mortality and CVDs [162, 163], which develops as a result of disorders of the renin-angiotensin-aldosterone system (RAAS), the sympathetic nervous system, and the immune system [164].

Research on the function of m^6^A modification in hypertension is still in its infancy. A study has unraveled that *FTO* rs9939609 is negatively associated with mean and diastolic blood pressure in male hypertension patients [165]. In another study, 1236 m^6^A-associated single nucleotide polymorphisms (SNPs) are nominally associated with blood pressure, and among them, rs7398833 in *CUX2* and rs13096477 in *SLC4A7* are the most significant [166], highlighting the potential effects of m^6^A in blood pressure regulation. Further, m^6^A RNA methylomes are altered and the abundance of m^6^A methylation is reduced in pericytes of spontaneously hypertensive rats [164]. Later on, another study described that the downregulation of FTO expression suppresses angiotensin II (AngII)-induced VSMC proliferation and inflammation by regulating the m^6^A methylation of *NR4A3* [111]. These results suggest the potential roles of m^6^A in hypertension-related vascular complications. Further exploration is warranted for how m^6^A modulates gene expression to trigger hypertension progression.

#### 3.1.3 m^6^A in AAD

Changed mechanical properties of the vessel wall with aging increase the fragility of artery and make it prone to aneurysm. Aortic aneurysm is the second most common disorder involving the aorta after AS, and its global burden remains high [167]. It is identified as a localized dilation of aorta attributed to acute factors (eg. trauma) or diseases (eg. hypertension). The large majority of aortic aneurysms are asymptomatic. However, progressive enlargement of aortic aneurysm increases the risk for aortic dissection, which can be life-threatening [168].

The m^6^A abundance remarkably increases in AAD in comparison with healthy aorta tissues and acts as a risk factor for aortic aneurysm rupture [169, 170]. Recent data from bioinformatic analyses suggest the potential roles of m^6^A in AAD [170-173]. VSMC dysfunction is a major contributor to the development and progression of aortic aneurysm and dissection [174]. Further evidence has revealed the possible involvement of m^6^A methylation in these diseases via regulating VSMC function [175-177]. Specifically, METTL3 promotes the formation of aortic aneurysm in mice through m^6^A-promoted miR-34a maturation and consequent downregulation of *SIRT1* in VSMCs [175]. Importantly, miR-34a has been found to stimulate VSMC senescence by *SIRT1* and enhance the expression of pro-inflammatory senescence-associated secretory phenotype [178]. METTL3-METTL14 complex promotes necroptosis and inflammation of VSMCs and progression of abdominal aortic aneurysms by mediating m^6^A modification of receptor-interacting protein 3 (*RIP3*) [179]. Moreover, the expression of METTL3 and FTO is increased in human aortic dissection tissues [176, 177]. METTL3 promotes ferroptosis of VSMCs by downregulating key ferroptosis regulatory proteins, solute carrier family 7 member 11 (*SLC7A11*) and ferroptosis suppressor protein 1 (*FSP1*); while the specific inhibitor of ferroptosis can alleviate the development and rupture of aortic dissection *in vivo* [177]. In another study, FTO mediates AngII-induced VSMC proliferation and migration, probably through m^6^A demethylation of *KLF5* [176]. Additionally, KIAA1429 and ALKBH5 oppositely affect aortic dissection progression through modulating VSMC proliferation by m^6^A-regulated pri-miR-143-3p maturation [180].

Overall, m^6^A methylation plays a great role in AAD by regulating the proliferation, migration, senescence, and programmed cell death of VSMCs. Future work on revealing the underlying mechanisms of the interplay between m^6^A and AAD are needed.

### 3.2 The roles of m^6^A in vascular aging-related heart diseases

Structural and functional alterations of the heart occur with aging, such as increased stiffness, myocardial hypertrophy, and cardiac dysfunction [181]. The role of m^6^A in the maintenance of cardiac homeostasis has been established [182, 183]. Emerging literature highlights the significance of m^6^A modification in regulating vascular aging-related heart diseases such as myocardial infarction (MI) and heart failure (HF) [6, 24].

#### 3.2.1 m^6^A in MI

The interplay between vascular aging and AS leads to the occurrence of MI. MI is a life-threatening disorder characterized by an abrupt drop in coronary blood flow, contributing to ischemia and, eventually, the loss of myocardium [184]. Recent studies have indicated that m^6^A presents multifaceted effects on MI and approaches targeting m^6^A exhibit potential for treatment of MI [24, 185].

m^6^A is an important player during the progression of MI. It is well-established that inflammation and cell death are key pathological alterations during the progression of MI [186]. It is reported that the expression and m^6^A level of hsa_circ_0029589 is decreased, while METTL3 expression is increased in macrophages from patients with acute coronary syndrome. Of note, IFN regulatory factor-1 (IRF-1) triggers macrophage pyroptosis and inflammation by inhibiting circ_0029589 via METTL3-mediated m^6^A modification [187]. Further, m^6^A-installed molecules can be transferred from ECs to cardiomyocytes and thus induce cardiac injury in MI. Mechanistically, hypoxia-induced METTL3 overexpression promotes m^6^A-mediated miR-503 maturation in ECs, which is transported to cardiomyocytes, eventually leading to mitochondrial dysfunction and cardiomyocyte death after MI [188].

m^6^A plays important roles in post-MI repair and regeneration by regulating cardiomyocyte proliferation, EC angiogenesis, and cardiac fibrosis. For instance, improving the expression of ALKBH5 remarkably decreases the infarct size, improves cardiac function, and enhances cardiomyocyte proliferation after MI in mice. This effect is mediated through ALKBH5-promoted YTHDF1 mRNA stability in an m^6^A-dependent manner in cardiomyocytes, which eventually contributes to increased Yes-associated protein (YAP) translation [189]. Moreover, ALKBH5 maintains EC angiogenesis during acute ischemic stress, which may be mediated by the demethylation of sphingosine kinase-1 (*SPHK1*) [190]. Similarly, implantation of METTL3-overexpression ECs improves post-ischemic neovascularization in MI mice by regulating the maturation of let-7e-5p and miR-18a-5p [191]. A recent MeRIP-seq analysis of heart samples from MI and control rats identified m^6^A-modified hub mRNAs and found their association with angiogenesis and apoptosis [192]. However, the role of these transcripts in MI pathologies needs to be further validated. In terms of cardiac fibrosis, METTL3 exhibits a pro-fibrotic role in the myocardium after MI. METTL3 silencing attenuates MI-induced cardiac fibrosis *in vivo* through the inactivation of cardiac fibroblasts mediated by m^6^A methylation of fibrosis-related genes such as small mothers against decapentaplegic homolog (*SMAD*) *2/3* [193].

#### 3.2.2 m^6^A in HF

HF is defined as a clinical syndrome consisting of symptoms and/or signs raised by structural or functional abnormality of ventricular filling or cardiac ejection [194]. Vascular aging-related reduction of coronary blood flow, AS, and hypertension are important mechanisms underlying HF pathogenesis. This disorder is the leading cause of hospitalizations in the elderly with high morbidity and mortality [195, 196]. HF is associated with deregulated epigenetic processes and abnormal gene expression [197]. Especially, RNA m^6^A methylation has been well-documented to participate in the development of HF [185, 198].

m^6^A levels in human, pig, and mouse failing hearts are increased compared to those in normal controls [182]. Also, transcriptome profiling of m^6^A is changed in the HF mouse model and failing human hearts, and differentially methylated transcripts code for proteins mainly associated with cardiac muscle differentiation and metabolic processes [197]. FTO is downregulated in failing mammalian hearts and hypoxic cardiomyocytes, and FTO overexpression in failing mouse hearts alleviates ischemia-induced decrease in cardiac function by selectively demethylating cardiac contractile transcripts such as sarcoplasmic reticulum calcium ATPase 2a (*SERCA2a*) [182]; while mice with cardiomyocyte-specific knockout of FTO presents worsened cardiac function compared to control mice [197], suggesting the necessity of FTO for maintaining cardiac homeostasis. Further studies revealed the underlying regulatory mechanism of FTO on HF progression. Specifically, FTO mitigates cardiac dysfunction in pressure overload-induced HF mice through modulating glycolysis at least partially by removing phosphoglycerate mutase 2 (*PGAM2*) m^6^A and also modulating glucose uptake probably by regulating the protein kinase B (AKT)-glucose transporter type 4 (GLUT4) pathway [199].

In addition to FTO, METTL3 also participates in the process of HF. It is reported that overexpression of METTL3 facilitates cardiomyocyte hypertrophy both *in vitro* and *in vivo* largely through m^6^A methylation of mitogen-activated protein kinases, whereas cardiac-specific METTL3 knockout mice present structural and functional characteristics of HF with aging and stress [183].

HF with preserved ejection fraction (HFpEF) is a large subset of HF. Whereas, fundamental biological processes involved in this disturbance remain largely elusive. A recent study found that m^6^A regulators (e.g., METTL3, METTL4, KIAA1429, FTO, and YTHDF2) are differentially expressed and m^6^A landscape is changed in HFpEF patients and/or mice, suggesting that m^6^A may act on the development of HFpEF [200]. Further, exercise training has been shown to alleviate myocardial phenotypes in high-fat diet (HFD)-induced HFpEF mice with altered m^6^A patterns and decreased FTO expression. However, overexpression of FTO counteracts the beneficial effects of exercise in HFpEF mice by inducing myocyte apoptosis, myocardial fibrosis, and myocyte hypertrophy [201].

Collectively, these studies suggest that METTL3 and FTO control cardiac homeostasis in an m^6^A-dependent manner, while their dysregulation is involved in HF progression. Further efforts to probe the underlying mechanisms of m^6^A and its regulators on the development of HF will help to provide new insight into biomarker identification and therapy exploration for HF.

### 3.3 The roles of m^6^A in vascular aging-related encephalopathy

Similar to the mechanism of vascular aging-related kidney diseases, elevated pulse pressure induced by vascular aging promotes the structural and functional abnormality of cerebral microvessels. These alterations can ultimately lead to cerebrovascular diseases and cognitive impairment [202].

#### 3.3.1 m^6^A in stroke

Vascular aging is an important player in the pathogenesis of stroke [203]. Stroke remains the second leading cause of death worldwide. The incidence and fatality of stroke increased heavily in the past three decades, particularly among the elderly [204]. Increasing data suggest the role of m^6^A in the pathogenesis of stroke [205].

Mo et al. identified 310 m^6^A-SNPs that were nominally associated with ischemic stroke [206]. Further data suggest that stroke changes the m^6^A profile. After cerebral ischemia, m^6^A abundance is increased and m^6^A epitranscriptome is changed significantly in mouse cortex [207, 208]. Further, METTL3-catalyzed m^6^A methylation promotes the maturation of miR-335, which induces stress granule formation and attenuates the apoptosis of injury neuronal cells by targeting eukaryotic translation termination factor (*eRF1*) in the early stage of acute ischemic stroke [209]. Several other m^6^A regulators have also been found to play a role in stroke. For example, YTHDC1 attenuates post-ischemic brain injury by promoting *PTEN* mRNA degradation to increase AKT phosphorylation [210]. Interestingly, exogenous FTO substantially minimizes poststroke brain damage and neurobehavioral deficits *in vivo* [211]. circSCMH1 promotes vascular repair after stroke through FTO-regulated m^6^A methylation of phospholipid phosphatase 3 (*PLPP3*) [212]. Moreover, miR-421-3p presents anti-inflammatory effects in cerebral ischemia/reperfusion injury by targeting YTHDF1 which mediates m^6^A modification of *P65* mRNA to regulate its translation [213].

#### 3.3.2 m^6^A in AD

AD is a complex neurodegenerative disease and is the leading cause of dementia in the elderly. The pathological hallmarks of AD are amyloid plaques consisting of β-amyloid (Aβ) peptides and neurofibrillary tangles composed of hyperphosphorylated tau in the brain. Age-related vascular alteration is a possible pathogenic factor in AD progression [214].

Evidence suggests that m^6^A modification is associated with AD [215]. Decreased m^6^A levels are detected in brain tissues of aged mice and AD patients [216, 217]. Of note, plentiful AD-associated transcripts present dysregulated m^6^A methylation in the AD mouse models and subsequently affect their protein levels, which indicates the potential pathogenic role of m^6^A in AD progression [216-218].

Reduced neuronal m^6^A abundance and METTL3 expression are found in human AD brains [219, 220]. METTL3 in the insoluble fractions is positively correlated with the abundance of insoluble tau protein in human AD samples [219]. Of note, METTL3 knockout in the hippocampus leads to memory deficits, synaptic loss, and neuronal death, which might be mediated by m^6^A dysregulation of the cell cycle gene, *Cyclin D2* [220]. A recent study reported that METTL3 facilitates autophagic clearance of p-Tau in the Aβ-induced cell model of AD via m^6^A-mediated stabilization of STIP1 homology and U-box containing protein 1 (*STUB1*) [221]. The expression of activity-regulated cytoskeleton-associated protein (ARC) is reduced in AD patients and cell models, which functions as a key factor for AD [222]. Interestingly, METTL3 rescues Aβ-stimulated decrease in *ARC* expression through YTHDF1-recognized m^6^A methylation [223]. However, METTL3 ablation in monocyte-derived macrophages attenuates AD pathology in a mouse model induced by Aβ-injection [224]. Further experiments revealed that METTL3 deficiency decreases m^6^A modification of DNA methyltransferase 3A (*DNMT3A*) that subsequently affects alpha-tubulin acetyltransferase 1 (*ATAT1*) expression [224].

An early prospective cohort study suggests that the *FTO* AA genotype is associated with a higher AD risk [225]. Consistently, another study also identified genetic variations in the *FTO* gene that might contribute to AD risk, and detected reduced FTO expression in human AD brains, indicating the potential function of FTO in AD pathology [226]. A more recent study revealed that conditional knockout of FTO in the neurons decreases cognitive deficits in AD mice. Mechanistic experiments suggested that FTO induces tau phosphorylation through activating tuberous sclerosis complex 1 (TSC1)-mTOR signaling [227]. Whereas few studies focus on the role of FTO-mediated m^6^A demethylation in AD progression.

Emerging evidence revealed the association of HNRNPA2B1 with tau that mediates the development of tauopathy [228]. Mechanistically, HNRNPA2B1 serves as a connector between oligomeric tau (oTau) and m^6^A-modified RNAs under AD conditions, subsequently regulating stress response and protein synthesis. While knockdown of HNRNPA2B1 reduces tau-induced neurodegeneration by dampening the association of oTau with m^6^A transcripts [228].

Bioinformatic analysis found that IGF2BP2 is highly expressed in human AD brain tissues. Besides, gene modules related to IGF2BP2 are significantly enriched in AD-associated biological processes, such as cytokine-cytokine receptor interaction and the TGF-β signaling pathway. Importantly, a diagnostic model for AD based on IGF2BP2-related genes has been constructed and validated [229]. These results indicate the potential of IGF2BP2 and associated m^6^A methylation in AD diagnosis and therapy.

Although several researchers have reported the role of m^6^A regulators in AD, especially METTL3, FTO, HNRNPA2B1, and IGF2BP2, the specific underlying molecular mechanisms remain largely unknown. Thus, in-depth mechanistic studies are warranted to provide a better understanding of the function of m^6^A in AD [230].

### 3.4 The roles of m^6^A in vascular aging-related kidney diseases

#### 3.4.1 m^6^A in CKD

Chronic kidney disease (CKD) refers to chronic abnormalities of renal function and/or structure, leading to a marked global disease burden [231]. Renal function progressively declines with aging. Vascular aging is significantly associated with CKD development [232]. Vascular aging-related artery stiffness can increase pulse pressure; its wave penetrates deeper into susceptible renal microvasculature, contributing to renal microvascular damage that may ultimately promote renal dysfunction and the development of end-stage renal disease [233].

Accumulating data indicate that m^6^A plays an important role in the pathogenesis of CKD. Renal fibrosis is a hallmark and common outcome in a variety of progressive CKD [234]. m^6^A may modulate renal fibrosis by targeting ncRNAs. For instance, METTL3-catalyzed m^6^A modification promotes renal fibrosis by promoting miR-21-5p maturation. Further experiments recovered that miR-21-5p promotes inflammation probably through the activation of Sprouty RTK signaling antagonist 1 (SPRY1)/extracellular signal-regulated kinase (ERK)/NF-κB pathway [235]. Another study reported that METTL3 modifies m^6^A methylation on long-non-coding RNA (lncRNA) MALAT1 to upregulate MALAT1 expression, which thereby promotes transforming growth factor β1 (TGF-β1)-induced renal fibrosis through miR-145/FAK signaling [236]. Moreover, Li et al. found that FTO promotes renal epithelial-mesenchymal transition (EMT) and inflammation response, which may be mediated by regulating m^6^A demethylation of lncRNA GAS5 [237]. These results provide a better understanding of novel mechanisms of m^6^A in CKD, which helps drug development for renal fibrosis.

Emerging evidence has also uncovered the important role of m^6^A in diabetic nephropathy (DN) progression. Lan et al. reported that WTAP induces *NLRP3* m^6^A methylation to mediate NLRP3 inflammasome activation in an IGF2BP1-dependent manner, further regulating cell pyroptosis and inflammation in DN models [238]. A recent study demonstrated that FTO expression is decreased in DN patients. Overexpression of FTO exerts protective effects during the pathogenesis of DN by increasing the expression of suppressors of cytokine signaling 1 (*SOCS1*) to attenuate inflammation response and kidney injury [239].

Vascular calcification is recognized as a common complication and marker of higher mortality and cardiovascular events in CKD patients [240, 241]. Previous research has established the role of m^6^A in regulating vascular calcification. Specifically, METTL14-dependent m^6^A motivates VSMC osteogenic conversion stimulated by indoxyl sulfate, possibly through methylating *Klotho* and inducing its degradation [242].

In summary, m^6^A methylation might affect the development of CKD by regulating the expression of genes involved in inflammation, EMT, fibrosis, and vascular calcification.

## 4. m^6^A as a Clinical Indicator

Vascular aging is an important cause of organ aging and a common pathogenesis of various chronic diseases. Its related diseases are highly harmful and have become a global challenge [243]. Currently, there is a lack of effective treatment options to reverse the progression of vascular aging. Therefore, further research is urgent to develop optimal clinical indicators.

Biomarkers refer to biological indicators that are objectively measured for the detection or evaluation of physiological processes, pathological processes, and the effectiveness of therapeutic approaches [244]. Although significant advances have been achieved in research on vascular aging mechanisms as stated above, effective methods for early identification of vascular aging remain limited. There is a growing need to develop novel biomarkers of vascular aging with simple operation, high sensitivity, strong specificity, and low cost. Biomarkers measurable in biofluids are potentially efficient, sensitive, and easily accessible indicators for the early detection of vascular aging-related diseases. Inflammatory factors such as C-reactive protein (CRP), interleukin-6 (IL-6), IL-1 receptor antagonist (IL-1Ra), and oxLDL, along with other circulating biomolecules like fibroblast growth factor 21 (FGF21), Fibulin-1, and miRNAs, have been reported as potential biomarkers of vascular aging. Additionally, the number and senescence of endothelial progenitor cells (EPCs) and senescence of immune cells such as macrophages, T cells, and B cells have been identified as circulating biomarkers of vascular aging as well [181]. Emerging evidence has revealed that epigenetic alterations including m^6^A modification during aging are closely linked with vascular aging [181, 245]. A fast response to environmental stimuli underlies the potential of m^6^A as the biomarker for the diagnosis of vascular aging and its related diseases. Some literature has reported the diagnostic and prognostic potential of m^6^A and its regulators in aspects of these conditions including coronary artery disease (CAD), HF, stroke, and kidney disease.

Decreased m^6^A abundance was measured in peripheral blood mononuclear cells (PBMCs) obtained from CAD patients compared to controls, where differentially methylated genes were revealed to participate in the pathogenesis of AS [246]. Of note, a diagnostic model of acute MI was established based on m^6^A-related genes, including FTO, WTAP, YTHDC1, IGFBP3, and CBLL1 [247]. Moreover, a genome-wide association study suggests that *WTAP* SNP is significantly associated with MI progression [248].

Reduced m^6^A abundance was observed in blood samples obtained during reperfusion in MI patients who developed HF four months after MI compared to those without HF [249]. Several m^6^A regulators (METTL3, METTL4, KIAA1429, FTO, and YTHDF2) were upregulated in PBMCs derived from HFpEF patients compared with the health group [200]. More efforts should be made to explore the feasibility of m^6^A as a novel biomarker in HF.

Additionally, the link between m^6^A-SNPs and the risk of ischemic stroke has been identified [250]. Eighty-four local genes (containing 87 m^6^A-SNPs) were detected to be differentially expressed in the peripheral blood from ischemic stroke patients, suggesting these m^6^A-SNPs as functional polymorphisms and new genetic biomarkers for ischemic stroke susceptibility [250].

Aside from circulating m^6^A, urine m^6^A has also been identified as a candidate biomarker in vascular aging-related kidney diseases. DN patients exhibit a marked reduction in urine m^6^A abundance compared to type 2 diabetes and normal glucose-tolerant cohorts. In particular, the levels of urine m^6^A decrease progressively as the disease worsens [251].

**Figure 5.**
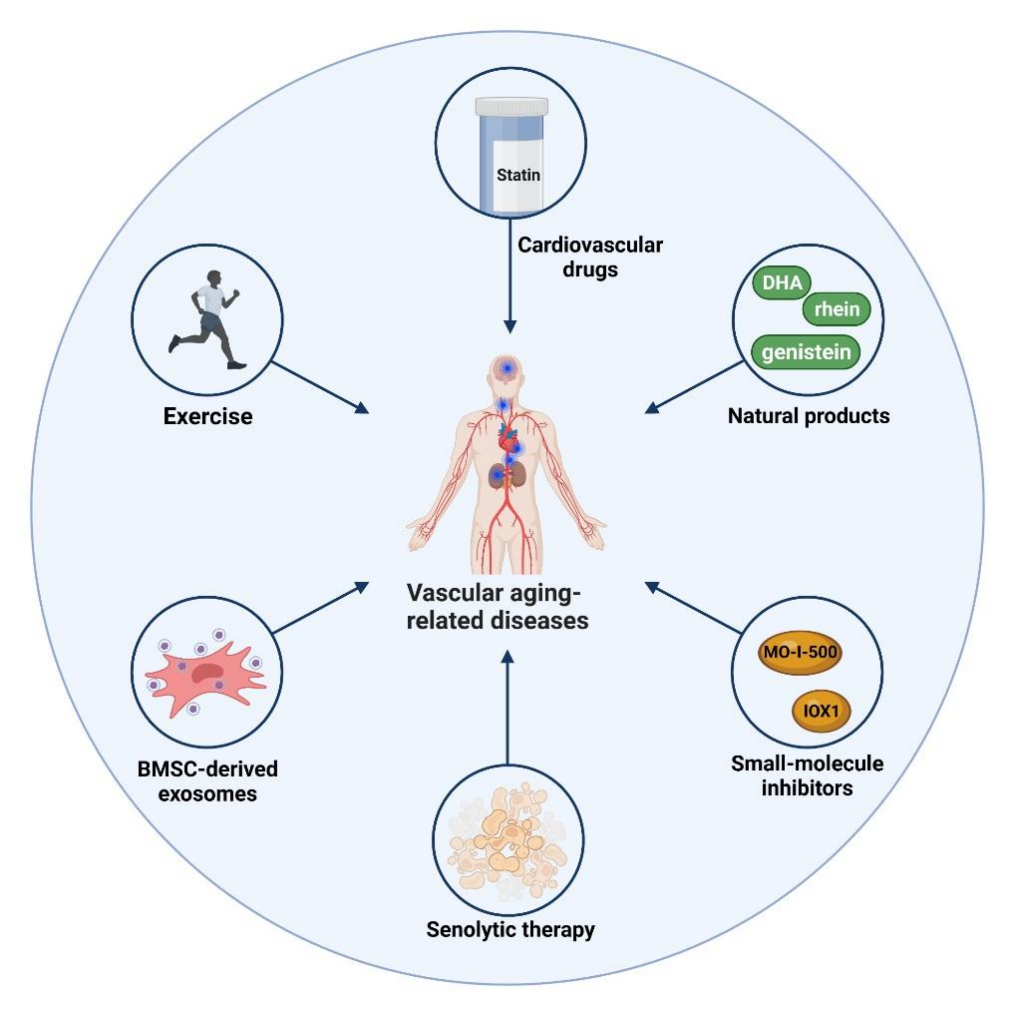
**m^6^A-associated therapies for vascular aging-related diseases**. m^6^A-associated anti-aging approaches present protective effects in vascular aging-related diseases, including exercise, senolytic therapy, BMSC-derived exosomes, cardiovascular drugs, natural products, and small-molecule inhibitors targeting m^6^A regulatory proteins. This figure was created with the aid of Biorender (https://biorender.com/). DHA: dihydroartemisinin; BMSC: bone marrow mesenchymal stem cell.

Further experimental and clinical research is required to confirm the utility of m^6^A methylation as a clinical indicator for vascular aging-related diseases. Therefore, future exploration should emphasize the evaluation of the relation between m^6^A and traditional clinical indicators of these disorders such as myocardial enzyme in MI, and N-terminal pro-brain natriuretic peptide in HF [252].

## 5. Therapeutic Potentials: Targeting m^6^A for Clinical Benefit

Healthy lifestyles (e.g., physical exercise, smoking cessation, certain diet regimens) are considered the easiest and most fundamental strategy in preventing vascular aging-related diseases. However, drugs targeting key aging-related molecular and cellular changes are still promising clinical therapies for these diseases. Existing clinical studies have observed the potential preventing effects of some clinically available drugs (e.g., statins, renin-angiotensin system inhibitors, and metformin) on vascular aging phenotypes during their administration for other medical conditions [4, 83]. Nonetheless, there are no medications developed specifically for the prevention and treatment of vascular aging. In recent years, multiple therapeutic approaches based on epigenetic alterations during aging have been developed for the aging process and age-related diseases [245]. Of note, aberrant m^6^A modification has been implicated in various molecular and cellular processes involved in vascular aging by regulating gene expression, contributing to the progression of related diseases. Therefore, targeting dysregulated m^6^A and its regulatory proteins provides potential therapeutic methods for various vascular aging-related diseases. Promising results have been obtained through research in this area (Fig. 5).

Recent data have described the protective effects of exercise on vascular aging-related diseases. Yang et al. reported that exercise attenuates endothelial pyroptosis and AS by downregulating METTL14 and m^6^A methylation of lncRNA NEAT1 [253]. Additionally, exercise training can alleviate HFpEF phenotypes by altering m^6^A modification patterns in mice [201].

Bone marrow MSC (BMSC)-derived exosomes exhibit promising therapeutic roles in various human diseases [254]. Emerging evidence suggests that BMSC-derived exosomal KLF4 promotes lncRNA-ZFAS1 expression to inhibit m6A methylation of dynamin-related protein 1 (*DRP1*) by targeting FTO, therefore mitigating mitochondrial dysfunction and ischemic stroke [255].

Moreover, a recent study reported that senolytic therapy of the combination of dasatinib and quercetin can attenuate LPS-induced EC senescence by upregulating YTHDF2 which destabilizes mitogen-activated protein kinase kinase 4 (*MAP2K4*) and mitogen-activated protein kinase kinase kinase kinase 4 (*MAP4K4*) mRNAs [256].

Traditional cardiovascular drugs such as classic lipid-lowering drugs, statins, can reduce FTO protein level and exerts protective effects on ECs by attenuating inflammation and increasing NO production. FTO attenuated statin-mediated effects on ECs by targeting *KLF2* and endothelial NOS (*eNOS*) through m^6^A demethylation and YTHDF3-mediated stabilization [257].

More than that, natural products from traditional medicine could be used as a chemical library for m^6^A-targeting anti-aging drug discovery. Recent evidence has identified some natural products that exhibit activating or inhibitory effects on m^6^A regulatory proteins (e.g., FTO and ALKBH5) and have potential therapeutic effects in various vascular aging-related diseases. For example, rhein, an anthraquinone concentrated in Rheumrhabarbarum, has been identified as a reversible and competitive inhibitor of FTO [20]. Inhibition of m^6^A demethylation through rhein treatment has partially rescued neurodegenerative changes induced by METTL3 knockdown [220]. Dihydroartemisinin, a first-line antimalarial drug originated from the natural small-molecule compound artemisinin, has also been shown to block FTO expression, leading to inhibiting AngII-regulated VSMC proliferation and inflammation by increasing *NR4A3* m6A methylation [111]. These results indicate the potential therapeutic role of dihydroartemisinin in hypertension-related vascular complications. Moreover, genistein, an isoflavone in soybean products widely used as a dietary supplement, has been found to elevate ALKBH5 expression and m^6^A levels. Genistein alleviates renal fibrosis by restoring ALKBH5 to regulate epithelial-to-mesenchymal transition [258].

Further, some pre-clinical experiments suggest that small-molecule inhibitors targeting dysregulated m^6^A regulators (e.g., FTO and ALKBH5) have potential therapeutic benefits in several vascular aging-related disorders. 5-carboxy-8-hydroxyquinoline (IOX1), a broad-spectrum inhibitor of most 2-OG oxygenases, can significantly repress ALKBH5 activity in a cofactor 2-OG competitive manner [259]. Since ALKBH5 plays a critical regulatory role in acute MI, IOX1 has been investigated for its therapeutic potential in treating acute MI. Notably, IOX1 was loaded onto bioengineered ferritin nanocage that can selectively target dying cells in the infarct territory. When administrated to the acute MI model, this nanocage improves cardiac function and minimizes the infarct area [260]. Given that disrupted m^6^A signaling is believed to play a role in AD pathogenesis, MO-I-500, a newly developed pharmacological inhibitor of FTO, has been revealed to promote cell survival and inhibit mitochondrial dysfunction in streptozotocin-treated astrocytes [261]. These findings suggest the therapeutic potential of MO-I-500 in AD.

The above findings suggest the potential therapeutic value of m^6^A-associated strategies for vascular aging-related diseases. These strategies include exercise, senolytic therapy, BMSC-derived exosomes, as well as targeting m^6^A regulatory proteins through cardiovascular drugs, natural products, and small-molecule inhibitors. Further research is required to explore the underlying mechanisms and translate these findings into clinical practice.

## 6. Patient-Centered Considerations

Patient-centered considerations are needed to improve outcomes, with a particular focus on the elderly population who often exhibit more complex care needs compared to younger counterparts [262, 263]. In-depth understanding of the intricate mechanisms that underlie diseases is crucial for clinician to customize interventions within a patient-centered framework [262].

In the context of patient-centered considerations, incorporating the knowledge of m^6^A modification into the management of vascular aging-related diseases can provide valuable insights. First, understanding the role of m^6^A in the pathophysiology of vascular aging and related diseases is helpful to strengthen clinical reasoning. By clarifying the underlying molecular mechanisms, clinicians can deepen their understanding of the disease process and identify potential therapeutic targets. This helps guide treatment decisions and tailor interventions for individual patients. Second, detecting the m^6^A landscape can guide patient subgrouping to obtain more targeted interventions. The distinct m^6^A patterns among individuals may be related to disease progression and treatment response differences [185, 264]. By stratifying patients according to the m^6^A landscape, clinicians can identify subgroups that may benefit from certain treatments, allowing them to develop personalized interventions [265]. Finally, m^6^A-associated biomarkers can be useful for personalized clinical diagnosis and treatment. By integrating technologies such as high-throughput technologies and bioinformatics analysis, clinicians can identify m^6^A-modified transcripts or m^6^A regulators as potential biomarkers to achieve early diagnosis, efficacy monitoring, and prognosis prediction [247, 248].

The integration of the knowledge of m^6^A into patient-centered care needs further research and validation in vascular aging-related diseases. However, a deeper understanding will help clinicians enhance their clinical reasoning, customize interventions through patient subgrouping, and apply m^6^A-related biomarkers to clinical diagnosis and treatment. These measures may contribute to a more individualized approach to the management of vascular aging-related diseases with a view to improving patient outcomes.

## 7. Challenges and Future Directions

Despite some advances and meaningful insights have been made in the investigation of m^6^A in vascular aging, challenges and exciting avenues for future research and clinical application remain. Further research is required to establish the precise function and potential mechanisms of m^6^A in this context, as well as its potential for clinical translation in the diagnosis and treatment of related diseases. Specifically, in-depth mechanistic studies on how m^6^A regulators coordinate to affect RNA fate and function and how specific m^6^A site is involved in different cellular signal pathways may bring a better understanding of its role in vascular aging. The development of more efficient and accurate methods to profile m^6^A RNA methylomes in various disease models can help to delineate condition-specific m^6^A patterns and identify precise methylation sites during vascular aging. Especially, ncRNA is also an important component of epigenetics in vascular aging [203]; however, studies are limited on its interplay with m^6^A modification during vascular aging progression. Further exploration is needed to enrich the knowledge in this area. Moreover, through integrative analysis of multi-omics data, a comprehensive analysis of the mechanisms underlying endothelial dysfunction and VSMC phenotypic transformation during vascular aging can be conducted. This approach is particularly useful for identifying novel potential targets for interventions in vascular aging. Currently, many small molecule inhibitors and activators of m^6^A regulatory factors have been developed, however, they are mostly explored in oncological disorders with poor specificity, efficacy, and safety [20]. Future work should be made to develop m^6^A inhibitors and activators with improved specificity, efficacy, and safety, and evaluate their function in vascular aging phenotypes. Epitranscriptome editing, similar to genome editing, has the potential to restore or remove functional m^6^A sites that are dysregulated or mutated in human diseases [23]. This approach might someday be applied to clinical practice in vascular aging-related diseases. In the future, clinical trials validating the diagnostic and therapeutic efficacy of m^6^A-related biomarkers and targeting therapy in vascular aging and related diseases could pave the way for new strategies for early detection and therapy.

## 8. A Multidisciplinary Approach: Collaborating for Progress

The complexity of vascular aging and related diseases is evident in the multifactorial influences (genetic factors, environmental factors), intertwined mechanisms, and multisystem involvement. Advancing the field of m^6^A research and its clinical application in these disorders necessitates a multidisciplinary approach. Collaboration among professionals such as clinicians, geneticists, molecular biologists, bioinformaticians, and pharmacologists enables to bridge the knowledge gap among different health care professionals, which helps to fully understand the effects of m^6^A on vascular aging. Collaboration is essential for revealing the epigenetic role in the disease process and provides a scientific basis, technical support, and possibility of novel diagnostic methods and targeted therapies.

## 9. Conclusion: Shaping the Future of Vascular Aging Care

m^6^A modification, as a key part of epitranscriptomics, has developed vigorously in the past decade. In this paper, we summarized the role of m^6^A methylation in vascular aging and related diseases and discussed its clinical prospect. The interaction between m^6^A modification and key pathways related to vascular aging bridges molecular insights and clinical realities. m^6^A exerts pleiotropic functions in various vascular aging-related diseases. m^6^A and its regulators detected in biofluid present diagnostic and prognostic potential in these conditions. m^6^A-associated anti-aging approaches exhibit potential protective effects in multiple vascular aging-related disorders. Moreover, integrating the knowledge of m^6^A into patient-centred care may help to improve clinical outcomes. While some progress has been made, there are still challenges ahead that demand further effort. Multidisciplinary collaboration is required to tackle these challenges and advance the field. With the continuous improvement and development of technology, the exploration of m^6^A can help to reveal new mechanistic insights into vascular aging process and may provide clinical diagnostic tools and therapeutic modalities for related diseases.

### Authors' contributions

Chen Li wrote the manuscript and generated the figures. Le Liu summarized the table and revised the manuscript. You-Shuo Liu and Shuang Li conceived the idea, guided the writing process, and supervised the manuscript. All authors have reviewed and approved the final manuscript.
